# P02.186. CAST (Centella asiatica selected triterpenes): stability, safety, and effect on diabetic neuropathy (DN)

**DOI:** 10.1186/1472-6882-12-S1-P242

**Published:** 2012-06-12

**Authors:** A Soumyanath, D Dimitrova, G Arnold, H Belding, N Seifer, N Le, J Lou

**Affiliations:** 1 Oregon Health & Science University, Portland, USA

## Purpose

The Ayurvedic nerve tonic herb Centella asiatica demonstrates potential neuro-regenerative properties in *in vitro* and *in vivo* models. The aims of the present study were to evaluate the safety and therapeutic effects of CAST (Indena®, Milan, Italy) in humans with DN, while monitoring the stability of CAST during the trial period.

## Methods

The stability of CAST was monitored throughout the study using reversed phase high performance liquid chromatography. CAST or placebo capsules were administered to 33 DN subjects, in a randomized double-blind, placebo-controlled study. The dose of CAST was escalated from 60 mg to 240 mg/day over the first 12 weeks, followed by a stable dose of 240 mg/day for the remaining 40 weeks. The primary outcome was total symptom score (TSS), while secondary outcomes were nerve conduction studies, neurological disability score (NDS) and qualitative sensory testing (QST).

## Results

CAST was stable (<10% change from starting values) at room temperature for the duration of the study. At baseline, there was no difference in outcome measures between treatment (n=15, 3F/12M) and placebo (n=18, 6F/12M) groups (TSS: 8.4±2.9 vs 8.3±2.5; combined sensory nerve action potential amplitude of sural and radial (SNAP): 18.8±8.9 vs 17.7±15.4 μV). At week 52, subjects in the treatment group had significantly lower TSS scores (6.4±0.4 vs 7.5±0.4, p<0.05) and higher SNAP (20.3±2.4 vs 14.8±2.2 μV, p<0.05) than the placebo group. Other outcome measures did not differ significantly between the two groups.

## Conclusion

CAST was stable and well tolerated by subjects at up to 240 mg per day. Compared to placebo, CAST significantly improved subjective DN symptoms (TSS score) and prevented deterioration in an objective measure of nerve conduction (SNAP). Current treatments for DN provide symptomatic relief rather than ameliorating disease progression. Multicenter trials are clearly warranted to further evaluate the role of CAST as a novel disease-modifying agent for DN.

